# Microalgae-mediated green synthesis of silver nanoparticles: a sustainable approach using extracellular polymeric substances from *Graesiella emersonii* KNUA204

**DOI:** 10.3389/fmicb.2025.1589285

**Published:** 2025-05-14

**Authors:** Jeong-Mi Do, Ji Won Hong, Ho-Sung Yoon

**Affiliations:** ^1^Integrated Blue Carbon Research Center, Advanced Bio-Resource Research Center, Kyungpook National University, Daegu, Republic of Korea; ^2^Department of Biology, College of Natural Sciences, Kyungpook National University, Daegu, Republic of Korea; ^3^BK21 FOUR KNU Creative BioResearch Group, School of Life Sciences, Kyungpook National University, Daegu, Republic of Korea

**Keywords:** microalgae, nanoparticles, biosynthesis, sustainability, tetracycline

## Abstract

Traditional nanoparticle synthesis relies on chemical and physical methods that often involve hazardous reagents, high energy consumption, and environmental toxicity. As a sustainable alternative, biological synthesis utilizes biomolecules in an eco-friendly manner to form nanoparticles. This study explores the green synthesis of silver nanoparticles (AgNPs) using extracellular polymeric substances (EPS) secreted by the microalga *Graesiella emersonii* KNUA204, highlighting the potential of microalgal biomolecules in nanotechnology. EPS-rich supernatant from *G. emersonii* enabled AgNP formation under light without the need for biomass pre-processing. The effects of culture age, pH (optimal at 10–11), and tetracycline as a secondary stabilizer were examined. Tetracycline accelerated AgNP formation in dark conditions but could not fully substitute light-induced reduction. The synthesized AgNPs and tetracycline-assisted AgNPs (Tetra-AgNPs) were characterized using UV-visible spectroscopy, EDX, XRD, FTIR, TEM, and Zeta potential measurements, confirming their crystalline, spherical, and moderately stable properties. Biological assays showed strong antibacterial activity at 10 μg mL^−1^, though Tetra-AgNPs did not outperform AgNPs or tetracycline alone, suggesting structural incorporation of tetracycline. Both AgNPs and Tetra-AgNPs showed similar antioxidant activity. These findings support the potential of *G. emersonii* KNUA204 for dual biomass utilization, integrating biofuel production with nanomaterial synthesis. Further optimization of EPS composition and biosynthesis conditions could enhance nanoparticle properties for biomedical and environmental applications, reinforcing microalgae as a platform for sustainable nanotechnology.

## 1 Introduction

Photosynthetic organisms, including algae, plants, and cyanobacteria, play a vital role in biotechnology due to their ability to synthesize diverse bioactive compounds. These organisms are widely cultivated for applications in food, pharmaceuticals, and cosmetics, as well as for their contributions to environmental sustainability through carbon sequestration and pollutant removal (Lin et al., 2019; Deviram et al., 2020). Among them, microalgae have garnered increasing attention as sustainable biofactories due to their high photosynthetic efficiency, rapid biomass accumulation, and adaptability to various cultivation systems. They have been extensively explored for biofuel production, carbon capture, and wastewater treatment, making them invaluable for green biotechnological applications (Hussain et al., 2021; Mehariya et al., 2021).

Microalgae serve as rich sources of high-value bioproducts, including carbohydrates, pigments, proteins, fatty acids, and bioactive secondary metabolites, which have applications in renewable energy, pharmaceuticals, and functional foods (Chu, 2017; Kusmayadi et al., 2021). Various biotechnological strategies—such as nutrient manipulation, light intensity control, and stress-induced metabolic shifts—have been employed to enhance the production of these compounds (Hu et al., 2021; Saejung and Chanthakhot, 2021; Rearte et al., 2024). In addition to intracellular metabolites, microalgae secrete extracellular polymeric substances (EPS), a class of high-molecular-weight biopolymers composed predominantly of polysaccharides, proteins, lipids, and uronic acids. EPS plays a crucial role in biofilm formation, cellular protection, and heavy metal sequestration, making it a key component in bioremediation and nanomaterial synthesis (Ozturk et al., 2014; Xiao and Zheng, 2016; Desmond et al., 2018). The production of EPS has been widely reported in microalgal species such as *Chlamydomonas reinhardtii, Chlorella vulgaris, Scenedesmus* sp., and *Graesiella emersonii* (Gongi et al., 2021; Trabelsi et al., 2016; Rahman et al., 2019; Huang et al., 2021; Koçer et al., 2021). Given its tunable properties and diverse bioactivities—including antimicrobial, antioxidant, and metal-chelating capabilities—EPS represents a promising bioresource for sustainable nanotechnology.

Nanotechnology has revolutionized medicine, electronics, and environmental remediation due to the exceptional physicochemical properties of nanomaterials, such as their high surface-area-to-volume ratio, enhanced reactivity, and tunable functionalization (Iravani et al., 2014). Among metallic nanomaterials, silver nanoparticles (AgNPs) have been extensively studied for their antimicrobial, anti-inflammatory, and catalytic activities, making them highly relevant for biomedical and environmental applications (Younis et al., 2022; Jangid et al., 2024). However, conventional AgNP synthesis methods rely on physical and chemical approaches that often involve toxic reagents, high energy consumption, and hazardous byproducts, raising environmental and health concerns (Bhushan et al., 2020). Green synthesis of AgNPs using biological agents offers a sustainable alternative by utilizing plant extracts, microbial secretions, and biopolymers such as EPS, which act as natural reducing and stabilizing agents (Iravani et al., 2014). Microalgae, in particular, are attractive bio-nanofactories due to their ability to secrete biomolecules that facilitate Ag^+^ reduction and nanoparticle stabilization.

Despite advancements in biological nanoparticle synthesis, the efficiency and control of particle formation remain key challenges. Recent studies have demonstrated that biomolecules such as proteins, polysaccharides, and even small-molecule antibiotics can influence nanoparticle properties by modulating their size, shape, and stability (Chen et al., 2019; Wei et al., 2020; Tubatsi et al., 2022; Radeghieri and Bergese, 2023). The integration of antibiotics into AgNP biosynthesis has gained interest due to the potential synergistic effects that enhance antibacterial efficacy, particularly against multidrug-resistant (MDR) bacterial strains (Gad El-Rab et al., 2021; Ramzan et al., 2022). Tetracycline, a broad-spectrum antibiotic, has been shown to act as both a reducing and stabilizing agent, influencing nanoparticle morphology and reactivity (Khurana et al., 2014). However, while antibiotic-functionalized AgNPs have exhibited enhanced bacterial penetration and biofilm inhibition (Bruna et al., 2021; Awadelkareem et al., 2023), their precise molecular interactions remain incompletely understood.

This study explores the green synthesis of AgNPs using EPS secreted by *Graesiella emersonii* KNUA204, a promising microalgal strain for sustainable nanotechnology due to its high EPS productivity, adaptability to nutrient-limited or wastewater-based cultivation, and scalability for industrial applications. The EPS-rich supernatant was employed as a natural reducing and stabilizing agent, facilitating the direct formation of AgNPs under light exposure without biomass pre-processing. To enhance nanoparticle formation, tetracycline was introduced as a secondary stabilizing agent, and its impact on AgNP synthesis was systematically examined under both light and dark conditions. Additionally, the influence of pH, culture age, and reaction environment on AgNP formation was investigated to optimize synthesis conditions for large-scale applications. The physicochemical properties of biosynthesized AgNPs and tetracycline-assisted AgNPs (Tetra-AgNPs) were characterized using UV-visible spectroscopy, Energy Dispersive X-ray spectroscopy (EDX), X-ray Diffraction (XRD), Fourier Transform Infrared spectroscopy (FTIR), Transmission Electron Microscopy (TEM), and Zeta potential measurements. Their biological functionalities were assessed through antioxidants and antibacterial assays to evaluate potential applications in medicine, environmental remediation, and sustainable biotechnology. By integrating microalgal EPS with antibiotic-assisted synthesis, this study provides new insights into the dual role of biological macromolecules in nanoparticle stabilization and antimicrobial enhancement. These findings contribute to the development of eco-friendly nanomaterials, offering a viable alternative to conventional synthesis methods and expanding the potential applications of microalgae in sustainable nanobiotechnology.

## 2 Materials and methods

### 2.1 Microalgae isolation and identification

A freshwater sample was collected from Ulleungdo Island, South Korea and inoculated into BG-11 medium for cultivation. The inoculated culture was maintained at 25°C under a 16:8 h light/dark cycle with an incident light intensity of 135 μmol m^−2^ s^−1^, using an orbital shaker set at 160 rpm. After 7 days of cultivation, the algal biomass was harvested via centrifugation and streaked onto BG-11 agar plates. The plates were incubated under the same conditions for an additional 7 days.

To obtain an axenic microalgal culture, a single colony was selected and re-streaked onto fresh BG-11 agar plates. The process was repeated until a pure culture was established. Morphological observation of the intact cells was conducted using an upright microscope (Axio Imager. A2, Carl Zeiss, Köln, Germany).

For species identification, genomic DNA was extracted using a DNA extraction buffer. The extracted DNA served as a template for PCR amplification of the 18S rRNA and internal transcribed spacer (ITS) regions using universal primers. PCR reactions were carried out using a thermocycler (TP350, TAKARA, Tokyo, Japan). The amplified sequences were analyzed using the NCBI BLAST tool to determine taxonomic affiliation. A phylogenetic tree was constructed using MEGA X software to further confirm species classification.

### 2.2 Microalgal cultivation and EPS extraction

Microalgae were cultivated in 100 mL of BG-11 medium within a 250-mL Erlenmeyer flask at an initial optical density at 600 nm (OD_600_) of 0.01. The culture was incubated at 25°C under a 16:8 h light/dark cycle with an incident light intensity of 135 μmol m^−2^ s^−1^, while continuously shaken at 160 rpm using an orbital shaker.

Once the culture reached the stationary phase, it was centrifuged at 1,516 × g, and the supernatant was transferred to a sterilized bottle. The EPS were extracted from the supernatant following the method described by Trabelsi et al., 2016.

### 2.3 Biochemical composition of EPS

Total carbohydrate content was quantified using the phenol-sulfuric acid method with modifications from Nielsen, 2010. Briefly, freeze-dried soluble EPS was hydrolyzed in 2.5 mL of 2 N H_2_SO_4_, vortexed, and heated at 100°C for 3 h. The hydrolyzed sample was cooled to room temperature and neutralized with Na_2_CO_3_, followed by dilution with ultrapure water (UPW) to a final concentration of 1 mg mL^−1^. After centrifugation at 1,516 × g for 5 min, the supernatant was transferred to a glass tube and mixed with 50 μL of 80% phenol solution and 5 mL of concentrated H_2_SO_4_. The mixture was incubated at room temperature for 10 min, followed by further incubation at 28°C. The absorbance was measured at 490 nm using a spectrophotometer.

Monosaccharide composition was determined following the NREL Laboratory Analytical Procedure. Lyophilized EPS was hydrolyzed by adding 250 μL of 72% H_2_SO_4_, incubating at 30°C for 1 h, and subsequently diluting with 7 mL of UPW. The hydrolyzed sample was autoclaved at 121°C for 1 h, then neutralized using concentrated CaCO_3_. After centrifugation, the supernatant was filtered and analyzed using high-performance liquid chromatography (HPLC) equipped with a refractive index detector (RID; Prominence, Shimadzu, Kyoto, Japan).

Uronic acid content was estimated using the carbazole assay. A Na_2_B_4_O_7_ solution was prepared by dissolving 0.9 g of Na_2_B_4_O_7_ in 10 mL of UPW, followed by the addition of concentrated H_2_SO_4_ and incubation overnight at room temperature. A carbazole solution was prepared by dissolving 100 mg of carbazole in 100 mL of absolute ethanol. Prior to analysis, the prepared solutions, samples, and D-galacturonic acid standards were cooled in an ice bath. Then, 1.5 mL of Na_2_B_4_O_7_ solution was added to 250 μL of either the sample or standard and heated at 100°C for 10 min, followed by cooling in an ice bath. Afterward, 50 μL of carbazole solution was added, and the mixture was further heated at 100°C for 15 min. The final solution was cooled to room temperature, and absorbance was measured at 525 nm.

Total protein content was measured using the Bradford assay. Prepared samples were mixed with Bio-Rad Protein Assay Dye Reagent Concentrate (Bio-Rad, Hercules, USA) according to the manufacturer's instructions. Bovine serum albumin (BSA; Sigma, St. Louis, USA) was used to generate a standard calibration curve. The absorbance of both samples and standards was measured at 595 nm.

Sulfate content was determined using the BaCl_2_-gelatin method. A BaCl_2_-gelatin solution was prepared by dissolving 0.5 g of gelatin in 100 mL of hot water (70°C) and storing the solution at 4°C overnight. After incubation, 0.5 g of BaCl_2_ was added. For the assay, 200 μL of either the prepared sample or K_2_SO_4_ standard solution was mixed with 3.8 mL of 4% trichloroacetic acid (TCA) and 1 mL of BaCl_2_-gelatin solution, followed by incubation at room temperature for 15 min. The absorbance was recorded at 360 nm.

### 2.4 Biosynthesis of AgNPs and tetra-AgNPs

To obtain the supernatant, *G. emersonii* KNUA204 cultures in the stationary phase were centrifuged at 1,516 × g for 5–10 min. The resulting supernatant was supplemented with AgNO_3_ solution at a final concentration of 0.5 mM and incubated under light (40.5 μmol m^−2^ s^−1^) and dark conditions at 25°C with continuous shaking (160 rpm). Successful AgNP synthesis was initially confirmed by a visible color change from transparent to light or dark brown, followed by UV-visible spectrophotometry using an X-ma 3200 spectrophotometer (Human Corporation, Seoul, Korea). For the synthesis of tetracycline-modified AgNPs (Tetra-AgNPs), different concentrations of tetracycline (10, 20, 30, 40, and 50 μg mL^−1^) were added to the reaction mixture and incubated under the same conditions as AgNP synthesis. Extracellular polymeric substances (EPS) in the supernatant served as the primary reducing and stabilizing agents in the green synthesis of AgNPs. Tetracycline was introduced as a secondary additive to assess its potential auxiliary role, particularly under dark conditions where light-dependent reduction is limited. To evaluate the effect of pH on AgNP synthesis, the pH of the microalgal supernatant was adjusted to 5, 6, 7, 8, 9, and 10 prior to the addition of AgNO_3_, and syn-thesis efficiency was analyzed via UV-visible spectrophotometry.

### 2.5 Physical characterization of AgNPs and tetra-AgNPs

The crystallinity of the biosynthesized nanoparticles was analyzed using XRD on an EMPYREAN diffractometer (Malvern Panalytical, Malvern, U.K.). The diffraction patterns were recorded over a 2θ range of 0° to 80°, employing Cu Kα radiation at a generator voltage of 40 kV and a tube current of 30 mA.

To examine the functional groups present in the EPS, AgNPs, and Tetra-AgNPs, FTIR spectra were recorded in the range of 4,000–400 cm^−1^ using a Frontier FTIR spectrometer (PerkinElmer, USA) with the KBr pellet method.

The morphology of the biosynthesized nanoparticles was examined using TEM on a Titan G2 ChemiSTEM Cs Probe (FEI Company, USA). Samples were prepared by depositing a drop of the nanoparticle suspension onto a carbon-coated copper grid and allowing it to dry at room temperature. The obtained TEM images were analyzed to determine nanoparticle size distribution using ImageJ software.

Elemental composition was determined using EDX Spectroscopy, equipped with a 4 SDDs windowless Super-X detector. The Zeta potential and hydrodynamic size distribution of the nanoparticles were measured using a Zetasizer Nano ZS (Malvern Panalytical, Malvern, U.K.) to assess their colloidal stability and surface charge properties.

### 2.6 Functional and antimicrobial properties of biosynthesized nanoparticles

#### 2.6.1 Antioxidant activity (DPPH assay)

The antioxidant activity of biosynthesized nanoparticles and microalgal-derived EPS was assessed using the 2,2-diphenyl-1-picrylhydrazyl (DPPH) radical scavenging assay. The experiment was conducted under dark conditions with ascorbic acid as positive control. Samples and standard controls were prepared at varying concentrations (0.3–10 μg mL^−1^).

For the assay, 100 μL of each prepared sample was added to 1,900 μL of 0.3 mM DPPH solution, vortexed vigorously, and incubated for 30 min at room temperature. The absorbance was measured at 517 nm, and radical scavenging activity was expressed as a percentage using the equation:


$$\begin{array}{l} \%\text{ DPPH scavenging activity }=\text{ 100 }\times \;(\text{1 }-\;(\text{sampleabsorbance})/\\ \;(\text{absorbanceof}\;\text{control})). \end{array}$$

#### 2.6.2 Antibacterial activity

The antibacterial activity of biosynthesized nanoparticles was evaluated using the disc diffusion method against four bacterial strains obtained from the Korean Collection for Type Cultures (KCTC): *Bacillus spizizenii* KCTC 2023, *Bacillus cereus* KCTC 3062, *Staphylococcus pasteuri* KCTC 13173, and *Escherichi coli* KCTC 2571. Each bacterial strain was cultured in LB medium (Becton, Dickinson and Company, USA), and 100 μL of bacterial culture was spread onto solidified LB agar plates. Sterile 6 mm disks containing 1–10 mg mL^−1^ of the antibacterial agents were placed on the agar surface. The plates were incubated at 37°C overnight, and inhibition zone diameters were measured.

The antibacterial effect in liquid medium was assessed by inoculating 10 μL of bacterial cultures into LB medium containing different concentrations of antibacterial agents (0, 2.5, 5, and 10 μg mL^−1^). Cultures were incubated at 37°C with continuous shaking (200 rpm) overnight, and bacterial growth was monitored by measuring OD600. The bactericidal percentage was calculated as follows:


$$\begin{array}{l} \%\text{ Bactericidal percentage }=\text{ 100 }\times \;((\text{OD of control sample-OD}\\ \text{ of test sample})/(\text{OD of control }\\ \text{ sample})). \end{array}$$

#### 2.6.3 Nanoparticle internalization in bacterial cells

The concentration of internalized AgNPs and Tetra-AgNPs within bacterial cells was measured using Inductively Coupled Plasma Optical Emission Spectroscopy (ICP-OES, Optima 7300 & Avio 500, PerkinElmer, USA) following the method described by Abdellatif et al. (2021).

Bacterial strains were cultured at 37°C, then exposed to 10 μg mL^−1^ of AgNPs and Tetra-AgNPs and incubated for 24 h at 37°C. After incubation, bacterial cells were harvested by centrifugation (13,652 × g), and the pellets were digested in a nitric ac-id-perchloric acid (3:1 v/v) mixture at 50–70°C for 5 min. The digested samples were di-luted with ultrapure water (UPW) and filtered through a 0.2 μm membrane filter (Minisart syringe filter, Sartorius Stedim Biotech, Germany) before ICP-OES analysis.

## 3 Results

### 3.1 Identification of the microalgal isolated from ulleundo island

BLASTn analysis using 18S rRNA and ITS sequences showed 99.83% and 100% matching with the sequences of *G. emersonii* CCAP 211/11N and *G. emersonii* CCAP 211/8H, respectively. Intact spherical single cell was observed using light microscope (Supplementary Figures 1C, D) and these results were similar to the morphology of previously reported *G. emersonii* (Heidari et al., 2017). Phylogenetic trees constructed using 18S rRNA and ITS sequences are shown in Supplementary Figures 1A, B.

### 3.2 Biochemical characterization of EPS from *G. emersonii* KNUA204

During the stationary phase, *G. emersonii* KNUA204 altered the transparent BG-11 medium to a light-yellow color, indicating the secretion of extracellular compounds. To analyze the biochemical composition of these secreted compounds, EPS was concentrated via centrifugation and lyophilized for further characterization.

Carbohydrates constituted 49.56 ± 0.72% of the total EPS content, while proteins, sulfates, and uronic acids accounted for 12.54 ± 1.25%, 11.81 ± 0.16%, and 13.14 ± 1.22%, respectively. HPLC analysis of the monosaccharide composition identified galactose as 14.89 ± 0.70% of the total sugar content, while two unidentified monosaccharides made up 79.08 ± 3.69% and 6.02 ± 4.30%, respectively.

### 3.3 Biosynthesis of AgNPs using *G.emersonii* KNUA204 supernatant

**Figure 1:**
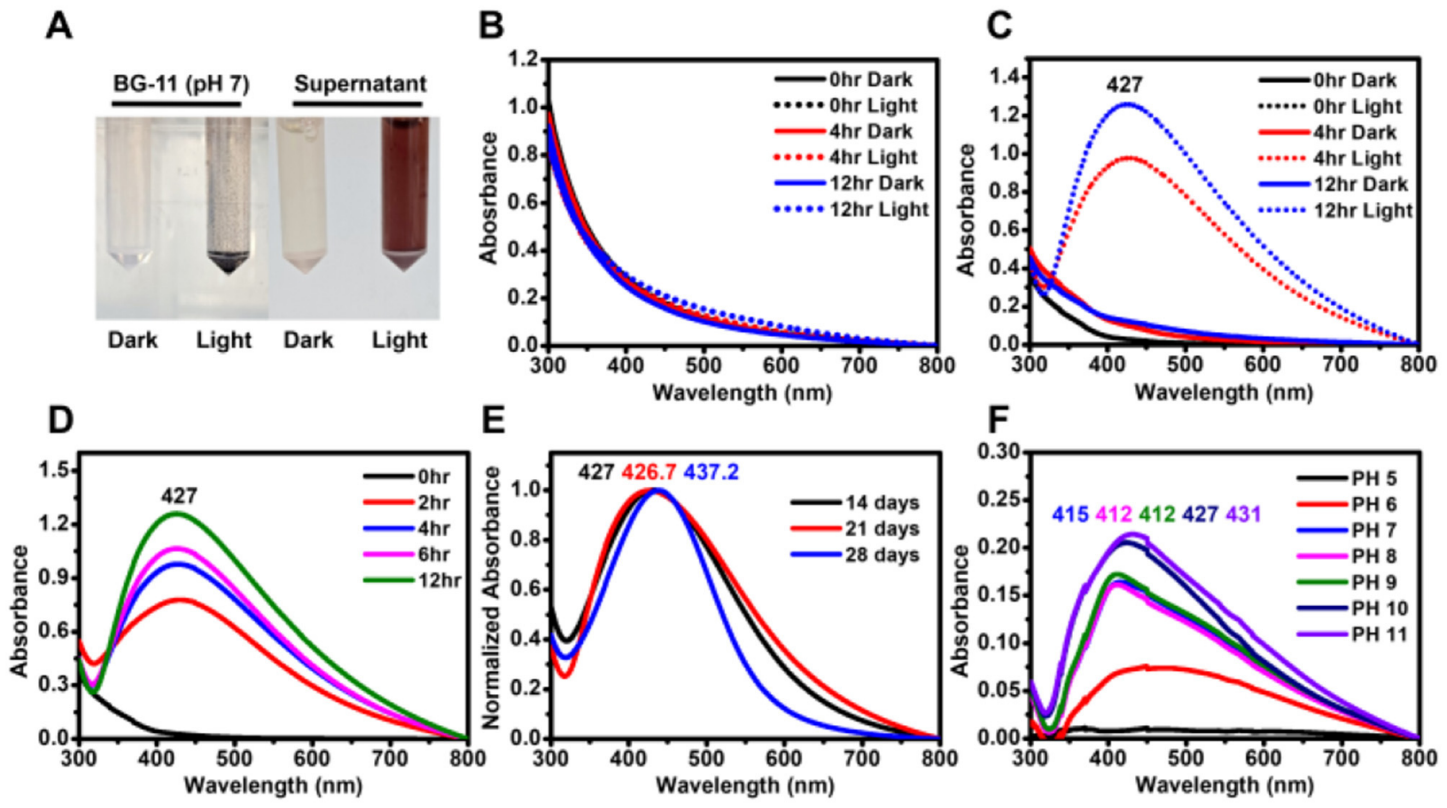
Biosynthesis of AgNPs using the supernatant of *G. emersonii* KNUA204. **(A)** Visual confirmation of AgNP formation in two mixtures: BG-11 + 0.5 mM AgNO_3_ and supernatant + 0.5 mM AgNO_3_, incubated under light and dark conditions. **(B)** Absorbance spectra of BG-11 + 0.5 mM AgNO_3_ in light and dark conditions, showing no significant peak formation. **(C)** Absorbance spectra of supernatant + 0.5 mM AgNO_3_, demonstrating AgNP formation under light exposure. **(D)** Effect of light exposure time on AgNP synthesis, showing increased absorbance with prolonged illumination. **(E)** AgNP synthesis using supernatants from 14-, 21-, and 28-day-old *G. emersonii* KNUA204 cultures, revealing spectral shifts with culture age. **(F)** Effect of pH variations on AgNP synthesis using the supernatant from a 14-day-old culture.

To determine whether the supernatant from *G. emersonii* KNUA204 culture could facilitate the biosynthesis of AgNPs, AgNO_3_ solution was added to the microalgal supernatant. After a few hours of light exposure, the reaction mixture exhibited a color change from transparent to brown, with a UV-visible absorption peak around 400 nm, confirming the successful synthesis of AgNPs (Figures 1A–C).

However, AgNP synthesis was not observed under dark conditions (Figure 1C), nor was any color change detected in AgNO_3_-treated BG-11 medium, indicating that the microalgal supernatant was essential for nanoparticle formation (Figure 1B). Prolonged light expo-sure resulted in higher absorbance values, suggesting an increase in nanoparticle concentration (Figures 1A–D).

To further investigate the effect of culture duration on AgNP synthesis, supernatants were collected from 14-, 21-, and 28-day-old microalgal cultures. The pH values of these supernatants were 10.11, 10.33, and 9.81, respectively. AgNPs synthesized using the 28-day supernatant exhibited a narrower absorption spectrum and a slight red shift in the maximum absorbance peak (Figure 1E).

Additionally, the effect of pH modulation on AgNP formation was examined. Acidification of the supernatant resulted in a blue shift in the absorption peak while maintaining a brown coloration, whereas alkalization led to a red-shifted peak and a dark brown color (Figure 1F and Supplementary Figure 2). These results suggest that pH variations influence the optical properties and characteristics of biosynthesized AgNPs.

### 3.4 Enhancement of AgNP biosynthesis by tetracycline

**Figure 2:**
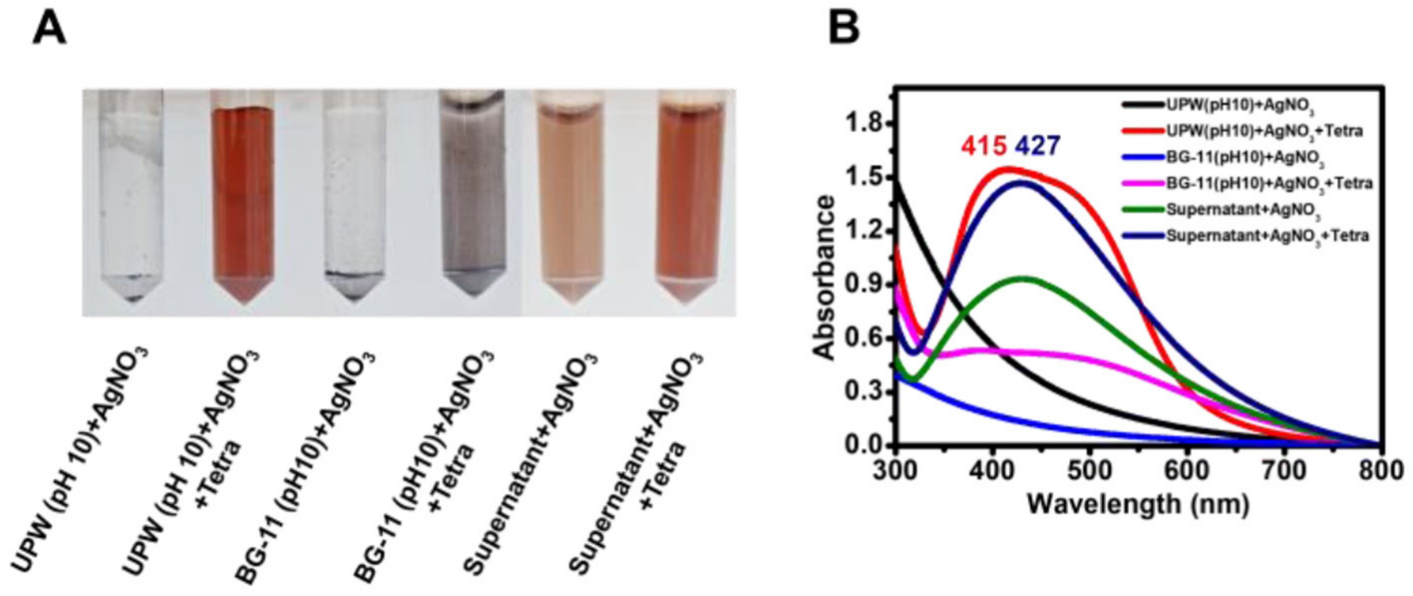
Synthesis of AgNPs using supernatant and tetracycline. **(A)** Color change in reaction mixtures after 3 h of light exposure, indicating AgNP formation under different conditions. **(B)** Absorbance spectra of the mixtures, showing variations in AgNP synthesis influenced by supernatant and tetracycline concentrations. Shifts in peak wavelength reflect variations in nanoparticle size, which may be influenced by reaction components and conditions.

To investigate the effect of tetracycline on AgNP biosynthesis, the antibiotic was added to a mixture of AgNO_3_ and microalgal supernatant. After 3 h of light exposure, the color intensity of the tetracycline-treated mixture was visibly higher than that of the control (without tetracycline-treated), and UV-visible spectrophotometry revealed a higher absorbance value in the tetracycline-treated reaction (Figure 2).

In an additional control experiment, AgNO_3_ in UPW at pH 10, with tetracycline, developed a brown coloration and an absorption peak at 415 nm, whereas no color change was observed in the absence of tetracycline. When pH 10-adjusted BG-11 medium was mixed with AgNO_3_ and tetracycline, larger aggregated dark particles were observed.

**Figure 3:**
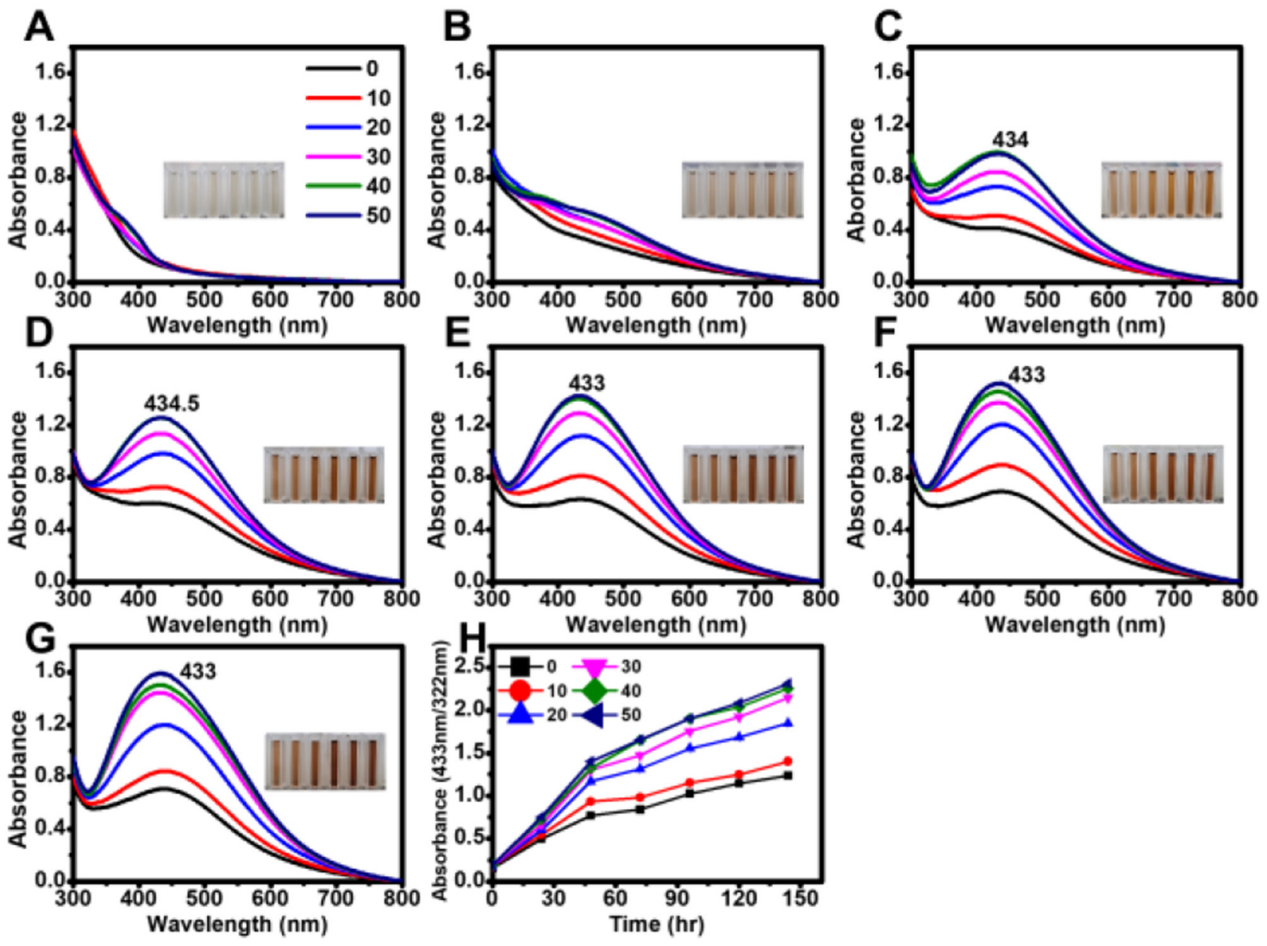
Effect of tetracycline on the biosynthesis of AgNPs under light exposure. Absorbance spectra of AgNPs synthesized under light with different tetracycline concentrations (0, 10, 20, 30, 40, and 50 μg mL^−1^) measured at various reaction times: **(A)** 0 min, **(B)** 30 min, **(C)** 60 min, **(D)** 90 min, **(E)** 120 min, **(F)** 150 min, and **(G)** 180 min. **(H)** Time-course analysis of AgNP biosynthesis, showing absorbance changes at 30 min intervals.

**Figure 4:**
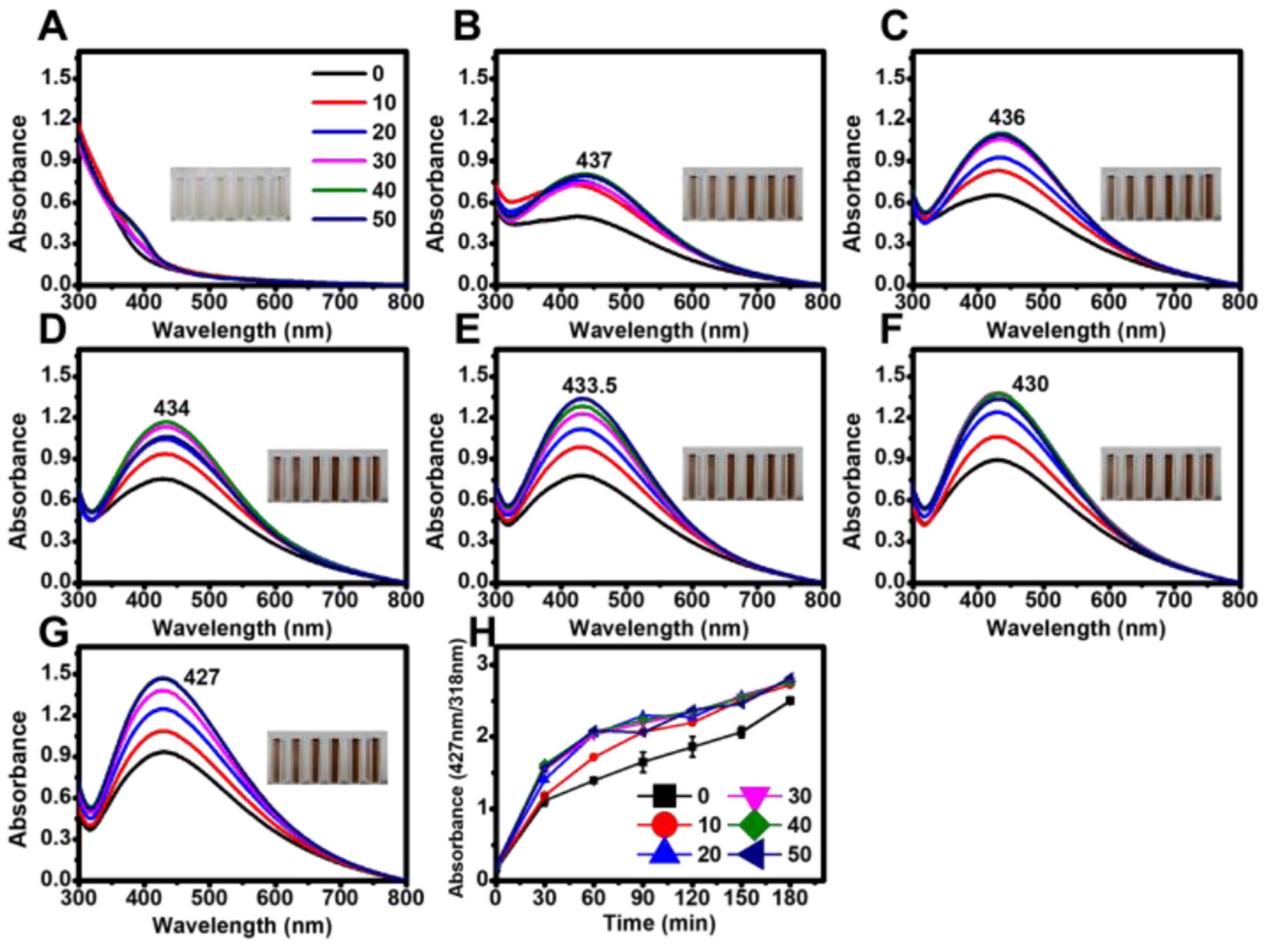
Effect of tetracycline on the biosynthesis of AgNPs under dark conditions. Absorbance spectra of AgNPs synthesized in the absence of light with varying tetracycline concentrations (0, 10, 20, 30, 40, and 50 μg mL^−1^) measured at different reaction times: **(A)** 0 h, **(B)** 24 h, **(C)** 48 h, **(D)** 72 h, **(E)** 96 h, **(F)** 120 h, and **(G)** 144 h. **(H)** Time-course analysis of AgNP biosynthesis, showing absorbance changes at 24-h intervals under dark conditions.

To determine whether tetracycline enhanced AgNP synthesis in the presence of microalgal supernatant, different concentrations of tetracycline (10–50 μg mL^−1^) were added to the AgNO_3_-supernatant mixture, and the reaction was monitored under both light and dark conditions. Although light accelerated AgNP synthesis, nanoparticles were also successfully formed in the dark (Figures 3, 4).

Under the tested conditions, maximum absorbance values were highly consistent at tetracycline concentrations of 30, 40, and 50 μg mL^−1^ in both light and dark conditions. In the presence of light, the absorption peak exhibited a blue shift with increasing reaction time (Figure 3), whereas no significant shift was observed in dark conditions (Figure 4).

### 3.5 Physical and chemical characterization of AgNPs and tetra-AgNPs

#### 3.5.1 Morphology and crystallinity

**Figure 5:**
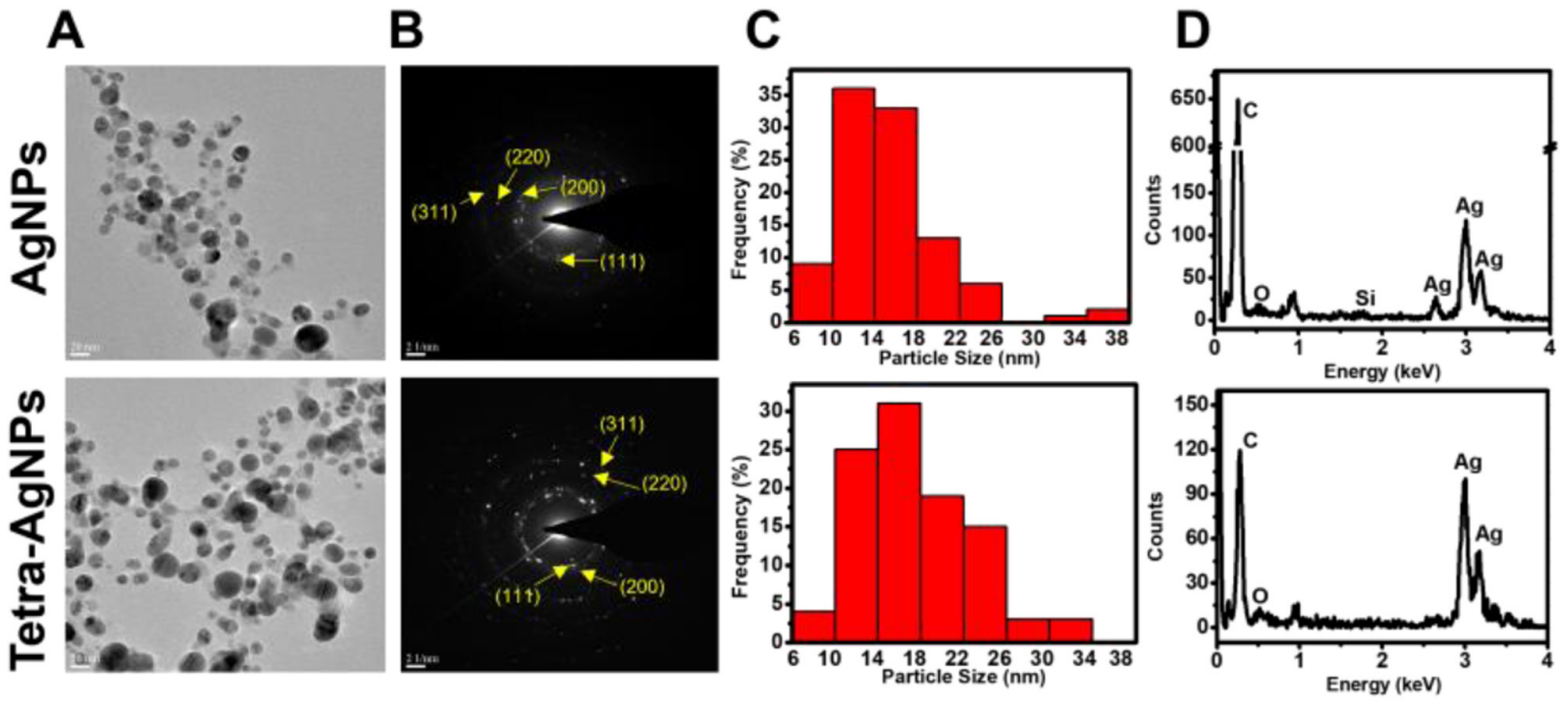
**(A)** Transmission electron microscopy (TEM) images showing the morphology and distribution of AgNPs **(top)** and Tetra-AgNPs **(bottom)**. **(B)** Selected area electron diffraction (SAED) patterns confirming the crystalline nature of both nanoparticles, with indexed diffraction rings corresponding to the (111), (200), (220), and (311) planes of silver. **(C)** Size distribution histograms of AgNPs and Tetra-AgNPs, indicating variation in nanoparticle size. **(D)** Energy-dispersive X-ray spectroscopy (EDX) analysis verifying elemental composition and confirming the presence of silver in both nanoparticle samples.

TEM analysis confirmed that the biosynthesized AgNPs and Tetra-AgNPs were spherical, with a size distribution ranging from 4 to 35.02 nm. The average diameters of AgNPs and Tetra-AgNPs were 15.40 ± 5.07 and 17.15 ± 5.35 nm, respectively (Figures 5A–C). EDX spectroscopy showed that silver (Ag) was the dominant element, with relative compositions of 29.92% in AgNPs and 66.60% in Tetra-AgNPs. Elemental mapping further verified the localized presence of Ag, confirming nanoparticle formation (Figure 5D and Supplementary Figure 3).

Selected Area Electron Diffraction (SAED) patterns exhibited bright concentric rings, indicating that both types of AgNPs were polycrystalline (Figure 5B). XRD analysis identified four major diffraction peaks at 2θ = 38.18, 44.20, 64.61, and 77.41° for AgNPs, while Tetra-AgNPs displayed peaks at 38.18, 44.17, 64.53, and 77.41°, corresponding to the (111), (200), (220), and (311) planes of silver, respectively (Supplementary Figure 4). The (111) plane was the dominant orientation in both samples. Additionally, AgNPs exhibited additional minor peaks, which were absent in Tetra-AgNPs, suggesting structural differences between the two types of nanoparticles. The preferred d-spacing of both AgNPs and Tetra-AgNPs was measured at 0.246 nm, aligning with the (111) plane of silver (Supplementary Figure 5).

#### 3.5.2 Functional group analysis (FTIR spectroscopy)

**Figure 6:**
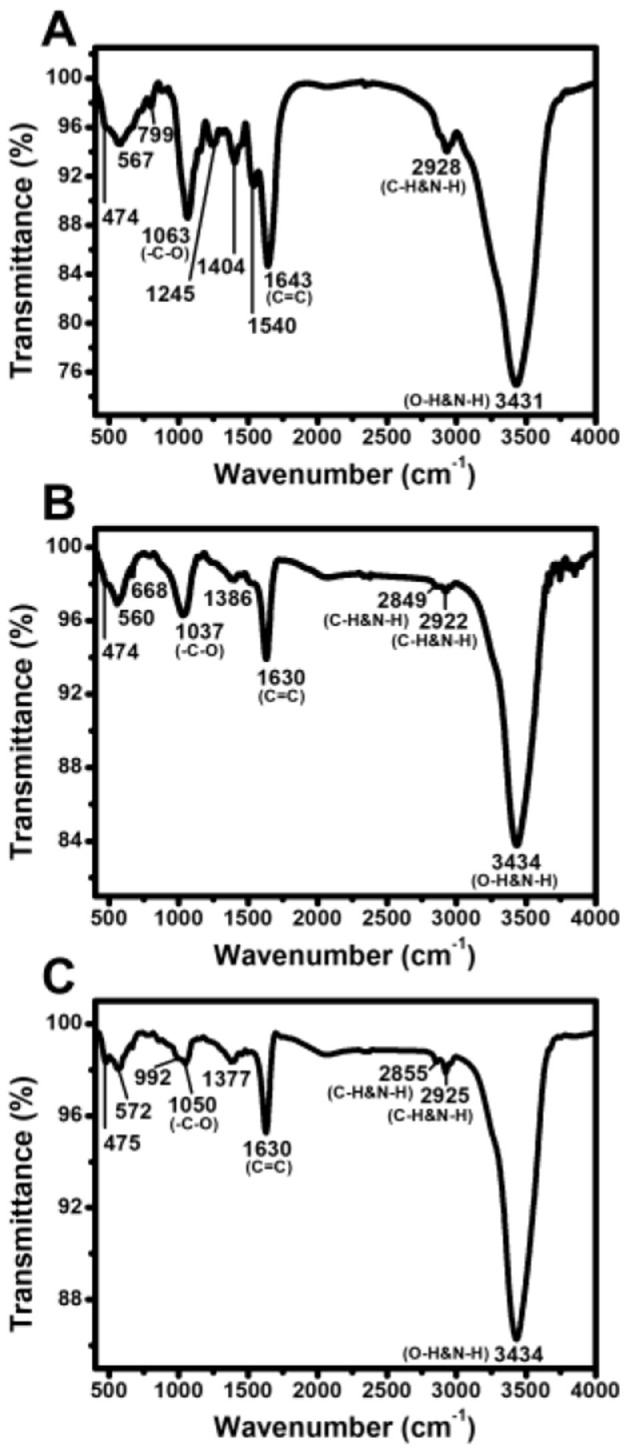
Fourier-transform infrared (FTIR) spectra of EPS, AgNPs, and Tetra-AgNPs. FTIR analysis of **(A)** extracellular polymeric substances (EPS), **(B)** biosynthesized silver nanoparticles (AgNPs), and **(C)** tetracycline-assisted silver nanoparticles (Tetra-AgNPs).

FTIR spectroscopy was conducted to identify the functional groups involved in AgNP stabilization. The spectral profiles of EPS, AgNPs, and Tetra-AgNPs exhibited 11, 9, and 9 peaks, respectively (Figure 6). The O-H and N-H stretching vibration bands observed at 3,431 cm^−1^ in EPS shifted to 3,434 cm^−1^ in both AgNP types, suggesting interactions with AgNPs. Peaks at 2,928 cm^−1^ in EPS, 2,849 and 2,922 cm^−1^ in AgNPs, and 2,855 and 2,925 cm^−1^ in Tetra-AgNPs were attributed to C-H stretching vibrations in hydrocarbon chains and N-H bending vibrations, indicating the possible in-volvement of proteins and polysaccharides in nanoparticle stabilization (Hamouda et al., 2019). A strong band observed at 1,643 cm^−1^ in EPS and 1,630 cm^−1^ in AgNPs and Tetra-AgNPs was associated with C=C and N-H stretching vibrations, suggesting the presence of amide and aromatic groups.

Additional peaks at 1,386 cm^−1^ in AgNPs and 1,377 cm^−1^ in Tetra-AgNPs were at-tributed to residual AgNO_3_ traces (Hamouda et al., 2019). The O-H peak at 1,154 cm^−1^, present in EPS, was absent in both AgNPs and Tetra-AgNPs, suggesting that hydroxyl groups were involved in AgNP reduction. Peaks at 1,063 cm^−1^ in EPS, 1,037 cm^−1^ in AgNPs, and 1,050 cm^−1^ in Tetra-AgNPs corresponded to C-O stretching, further confirming the role of polysaccharides in the stabilization process. Peaks in the 450–700 cm^−1^ range were at-tributed to bending vibrations of aliphatic groups, supporting the presence of biomolecules in AgNP stabilization.

#### 3.5.3 Stability and surface charge (zeta potential analysis)

**Figure 7:**
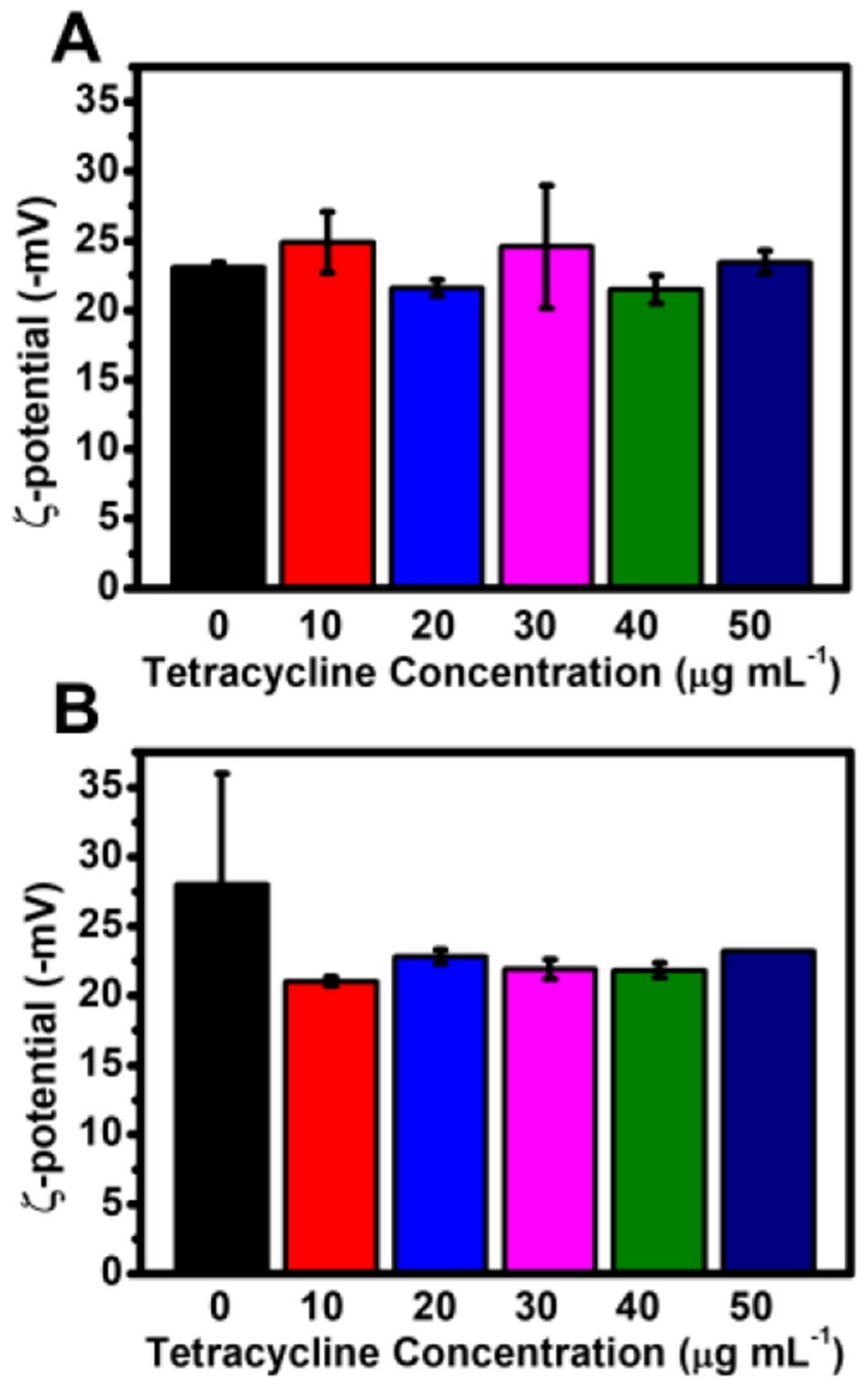
Zeta potential of AgNPs synthesized with varying tetracycline concentrations under light and dark conditions. **(A)** Zeta potential values of AgNPs synthesized under light conditions with different tetracycline concentrations. **(B)** Zeta potential values of AgNPs synthesized under dark conditions with different tetracycline concentrations. Error bars represent standard deviations.

To assess the stability of AgNPs synthesized with different tetracycline concentrations, Zeta potential (ζ-potential) measurements were conducted under both light and dark conditions (Figure 7). The ζ-potential values of AgNPs synthesized in light with tetracycline concentrations of 0, 10, 20, 30, 40, and 50 μg mL^−1^ were −23.07 ± 0.33, −24.87 ± 2.19, −21.60 ± 0.57, −24.57 ± 4.41, −21.50 ± 0.99, and −23.43 ± 0.83 mV, respectively. In dark conditions, the ζ-potential values measured at the same tetracycline concentrations were −27.97 ± 8.02, −21.03 ± 0.33, −22.80 ± 0.50, −21.90 ± 0.70, −21.83 ± 0.53, and −23.20 ± 0.08 mV.

Overall, these results indicate that tetracycline did not significantly alter the surface charge of AgNPs, and the nanoparticles maintained colloidal stability across all tested conditions.

### 3.6 Antioxidant and antibacterial activity of biosynthesized AgNPs

#### 3.6.1 Antioxidant activity

The antioxidant potential of biosynthesized AgNPs and Tetra-AgNPs was assessed using the DPPH radical scavenging assay. Both nanoparticles exhibited similar DPPH scavenging efficiencies of approximately 60%, indicating moderate antioxidant activity. However, their antioxidant efficiency did not exceed that of ascorbic acid, which served as the positive control (Supplementary Figure 6).

#### 3.6.2 Antibacterial activity

The antibacterial potential of biosynthesized AgNPs and Tetra-AgNPs was evaluated against Gram-positive bacteria (*Bacillus spizizenii, Bacillus cereus*, and *Staphylococcus pasteuri*) and Gram-negative *Escherichia coli* using both disc diffusion and liquid culture assays. In the disc diffusion assay, *B. spizizenii* exhibited the highest susceptibility to both AgNPs and Tetra-AgNPs, forming the largest inhibition zones (Supplementary Figure 8 and Table 1). However, inhibition zones for *B. cereus, S. pasteuri*, and *E. coli* were relatively smaller, indicating lower susceptibility to these nanoparticles (Supplementary Figure 7 and Table 1).

**Table 1:** Antibacterial activity of AgNO_3_, AgNPs, tetra-AgNPs, and tetracycline against different bacterial species, measured by the disc diffusion method.

**Antibacterial agents**	**Bacterial species/inhibition zone diameter (mm)**			
	***B. spizizenii***	***B. cereus***	***S. pasteuri***	***E. coli***
AgNO_3_	9.67 ± 0.24	10.17 ± 1.03	10.00 ± 0.71	9.33 ± 0.62
AgNPs	10.83 ± 1.03	8.17 ± 0.24	8.33 ± 0.47	7.83 ± 0.24
Tetra-AgNPs	10.17 ± 0.24	7.67 ± 0.47	7.83 ± 0.24	7.16 ± 0.24
Tetracycline	23.50 ± 1.22	20.67 ± 0.94	20.83 ± 0.62	13.30 ± 0.47

**Figure 8:**
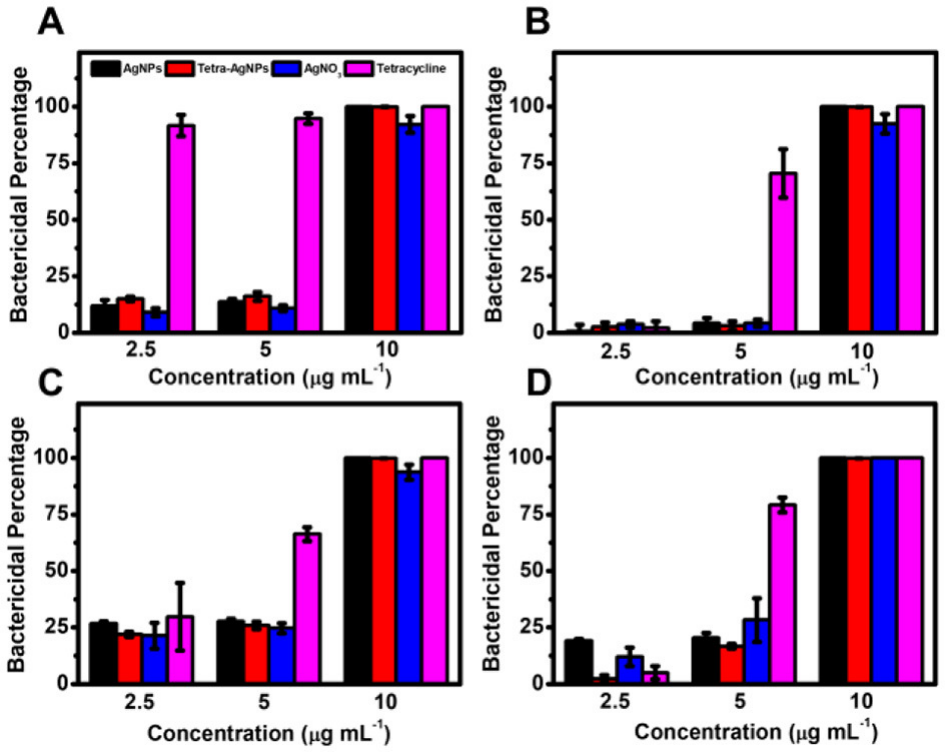
Antibacterial activity of AgNPs and Tetra-AgNPs in liquid culture. Bactericidal effects of AgNPs and Tetra-AgNPs against four bacterial strains: **(A)** *B. spizizenii*, **(B)** *B. cereus*, **(C)** *S. pasteuri*, and **(D)** *E. coli*. Bacterial growth inhibition was assessed by measuring optical density (OD600) after incubation with different antibacterial agents. Error bars represent standard deviations (*n* = 3).

In the liquid culture assay, bacterial growth was not significantly inhibited at AgNP concentrations of 2.5 and 5 μg mL^−1^ (Figure 8). However, at 10 μg mL^−1^, both AgNPs exhibited ~100% bactericidal activity against all four bacterial species, demonstrating dose-dependent antibacterial efficacy.

To confirm whether AgNPs and Tetra-AgNPs were internalized into bacterial cells, their intracellular concentrations were quantified using ICP-OES. Higher concentrations of both nanoparticles were detected in *B. cereus, S. pasteuri*, and *E. coli* compared to *B. spizizenii*, suggesting species-dependent nanoparticle uptake efficiency (Figure 8).

## 4 Discussion

The development of sustainable nanomaterials with optimized size, stability, and functionality remains a major challenge in nanotechnology. Biological synthesis using microalgae provides an eco-friendly alternative to conventional chemical and physical methods, offering a renewable source of reducing and stabilizing agents. Unlike synthetic reagents, microalgal biomolecules, particularly extracellular polymeric substances (EPS), allow for controlled nanoparticle formation with reduced environmental impact. The physicochemical properties of biosynthesized nanoparticles depend on the species-specific biochemical composition of microalgal metabolites (Patel et al., 2015; Moraes et al., 2021). This approach aligns with the increasing demand for green nanotechnology in biomedical and environmental applications. In this context, *G. emersonii* KNUA204 was selected as an optimal species for nanoparticle biosynthesis. This strain has demonstrated robust growth in wastewater-based cultivation systems and is known for its high yield of EPS, which are crucial for nanoparticle formation. Moreover, its potential for lipid accumulation makes it attractive for integrated biorefinery systems that combine biofuel and nanomaterial production.

Most previous studies on microalgae-derived nanoparticle synthesis have relied on harvested biomass, requiring additional steps such as boiling, homogenization, and extraction to obtain reducing agents (Trabelsi et al., 2016). However, this study demonstrates that EPS secreted by *G. emersonii* KNUA204 can act as natural capping agents, enabling direct biosynthesis of AgNPs using cell-free supernatant under light exposure. This method presents a novel and cost-effective strategy, allowing for the simultaneous utilization of microalgal biomass for other high-value applications such as biofuel production and pharmaceuticals while using culture supernatant for nanoparticle synthesis, enhancing resource efficiency (Mata et al., 2010).

The results suggest that *G. emersonii* KNUA204 is an optimal species for this approach due to its scalability and ability to grow in wastewater, such as vermicompost-treated water (Wen et al., 2016; Santhana Kumar et al., 2022). Additionally, its ability to accumulate lipids over an extended cultivation period enhances its value for bioenergy applications (Wen et al., 2016). As cultures aged, the concentration of soluble EPS in BG-11 medium increased, contributing to improved AgNP synthesis (Supplementary Figure 8). This suggests that older *G. emersonii* KNUA204 cultures hold greater potential for dual utilization, making them ideal for integrated biorefinery approaches.

The EPS composition of *G. emersonii* KNUA204 consisted mainly of carbohydrates, proteins, sulfates, and uronic acids, aligning with previous reports (Trabelsi et al., 2016). However, minor variations in EPS composition were observed, likely due to differences in cultivation conditions and strain origin. Unlike *Graesiella* sp. isolated from a 60°C hot spring, which exhibited a higher uronic acid content, the *G. emersonii* KNUA204 EPS in this study contained a lower uronic acid fraction, which may affect its metal-complexing capacity (Ozturk et al., 2014; Costa et al., 2021). The presence of anionic groups in EPS, particularly uronic acids, contributes to its ability to bind and stabilize nanoparticles, highlighting the role of EPS in facilitating AgNP biosynthesis. Interestingly, one unknown monosaccharide constituted the highest proportion in the EPS, suggesting the need for further characterization to understand its contribution to nanoparticle synthesis and stabilization.

Consistent with previous studies, the results confirm that light plays a crucial role in AgNP biosynthesis using EPS-rich supernatants (Patel et al., 2015; Rahman et al., 2019; Pandey et al., 2021). The biosynthesis mechanism can be divided into three stages: adsorption of Ag^+^ onto EPS in a light-independent manner, reduction of Ag^+^ to Ag^0^ as a light-dependent reaction, and stabilization of AgNPs via EPS in a light-independent step (Rahman et al., 2019). AgNPs synthesized using 28-day-old culture supernatants exhibited a narrow absorbance spectrum with a red-shifted maximum peak, suggesting increased particle size due to higher EPS concentration (Figure 2E and Supplementary Figure 8). Larger AgNPs are known to exhibit enhanced resistance to aggregation and higher biological activity (Bélteky et al., 2021). The pH of the supernatant also played a critical role in biosynthesis, with an optimal pH range of 10–11 (Figure 2F). This is similar to *Desmodesmus abundans* but higher than the optimal conditions reported for *Oscillatoria limnetica* and *Spirulina platensis* (Muthusamy et al., 2017; Hamouda et al., 2019; Mora-Godínez et al., 2020).

Antibiotic-functionalized AgNPs have been explored for enhanced antimicrobial activity. In this study, tetracycline facilitated AgNP synthesis, particularly under dark conditions, by increasing absorbance values relative to tetracycline-independent AgNPs (Djafari et al., 2016; Khan et al., 2019; Bruna et al., 2021). The broad absorbance spectrum with a shoulder at ~500 nm suggested the formation of mixed spherical and cuboidal AgNPs in sterile distilled water, whereas EPS-mediated AgNPs remained spherical (Restrepo and Villa, 2021). While tetracycline accelerated AgNP synthesis, it did not completely replace the light-dependent reduction process. Furthermore, although no morphological differences were observed in TEM images, FTIR analysis revealed spectral shifts in functional groups associated with hydroxyl, amide, and aromatic moieties, suggesting interaction of tetracycline with the AgNP surface. These findings imply that tetracycline molecules likely adsorbed onto the nanoparticle surface through non-covalent bonding rather than forming a core-shell encapsulation, contributing to colloidal stability without significantly altering particle morphology. The light-independent reaction proceeded at a much slower rate, indicating that tetracycline alone is not a sufficient alternative to light exposure for rapid AgNP formation (Figure 5). This may be due to the absence of the light-driven electron donation step, a critical factor in EPS-mediated biosynthesis (Rahman et al., 2019).

TEM and XRD analysis confirmed that AgNPs and Tetra-AgNPs were crystalline, predominantly spherical, and moderately stable. The presence of additional peaks in the AgNP XRD pattern may indicate impurities or organic capping molecules, which were absent in Tetra-AgNPs. The average diameter of Tetra-AgNPs was slightly larger than non-tetracycline-bound AgNPs (Figures 6A, C). The Zeta potential values of both AgNPs and Tetra-AgNPs ranged between ±20–30 mV, classifying them as moderately stable colloidal systems. While these values suggest moderate colloidal stability, we acknowledge that direct comparison with the zeta potentials of individual components, such as EPS and tetracycline alone, would offer deeper insights into their specific contributions. Although these individual measurements were not included in the current study, this limitation should be considered when interpreting the stabilization mechanism. The biosynthesized nanoparticles were internalized by bacterial cells, confirming their interaction with bacterial membranes and supporting their bactericidal mechanism (Liao et al., 2019).

Both AgNPs and Tetra-AgNPs displayed strong antibacterial activity at 10 μg mL^−1^, but Tetra-AgNPs did not exhibit enhanced efficacy compared to AgNPs or tetracycline alone. This suggests that tetracycline's A-ring, which is crucial for bacterial ribosome binding, was not fully exposed in Tetra-AgNPs, reducing its antimicrobial effectiveness (Tariq et al., 2018). Similarly, the antioxidant activity of AgNPs and Tetra-AgNPs remained comparable at approximately 60%, indicating that tetracycline incorporation did not enhance ROS scavenging properties (Kładna et al., 2012; Bedlovičová et al., 2020). These findings suggest that while tetracycline aids in nanoparticle stabilization and synthesis, it does not necessarily improve biological functionality when conjugated with AgNPs. This may be explained by the potential masking or steric hindrance of tetracycline's active sites (e.g., the A-ring), which are essential for its antimicrobial action. When adsorbed onto the nanoparticle surface, these sites may become less accessible for interaction with bacterial ribosomes. In addition, although increased colloidal stability is generally associated with higher surface activity, the actual antibacterial performance depends on both the availability and orientation of functional groups on the nanoparticle surface. Therefore, the conjugation of tetracycline, while beneficial for nanoparticle formation and dispersion, may compromise its bioactive function. To better contextualize these findings, a comparative summary of AgNPs synthesized using different microalgal species was compiled (Supplementary Table 1). While biosynthetic routes vary in terms of nanoparticle size, shape, stability, and antibacterial efficacy, the AgNPs produced in this study fall within the expected range and exhibit comparable or superior performance against selected bacterial strains. However, consistent with other studies, the conjugation of bioactive molecules such as antibiotics does not always result in improved biological outcomes, underlining the importance of structural accessibility and surface chemistry in nanoparticle design. These findings emphasize the importance of carefully balancing chemical stability and biological activity when designing antibiotic-functionalized nanoparticles.

This study demonstrates the feasibility of using microalgal EPS as a bioresource for sustainable AgNP biosynthesis, providing a novel cell-free approach that enhances nanoparticle production while preserving biomass for other applications. The dual utilization of microalgal supernatants and biomass aligns with biorefinery strategies, maximizing efficiency for biofuel production, nanomaterial synthesis, and wastewater treatment. Further research should focus on optimizing EPS composition, culture conditions, and nanoparticle functionality for applications in biomedicine, antimicrobial coatings, and environmental remediation. This study reinforces the potential of microalgae as a platform for eco-friendly nanotechnology, paving the way for scalable and industrially viable microbial nanomaterial production.

## 5 Conclusion

This study demonstrates that extracellular polymeric substances (EPS) from Graesiella emersonii KNUA204 enable the green synthesis of silver nanoparticles (AgNPs), offering a sustainable alternative to conventional methods. The EPS-rich supernatant facilitated AgNP formation under light, with older cultures and alkaline pH (10–11) enhancing nanoparticle size and stability. This cell-free biosynthesis maximizes resource efficiency by repurposing supernatants for nanomaterial production while preserving biomass for biofuel or other high-value applications. Tetracycline-assisted AgNPs (Tetra-AgNPs) were synthesized to explore antibiotic-mediated biosynthesis. Tetracycline accelerated AgNP formation in dark conditions but could not replace light-driven electron transfer in EPS-mediated synthesis. Both AgNPs and Tetra-AgNPs were crystalline, spherical, and moderately stable, displaying strong antibacterial activity at 10 μg mL^−1^, though Tetra-AgNPs did not surpass AgNPs or tetracycline alone. Similarly, ROS scavenging activity remained comparable, indicating no antioxidant enhancement from tetracycline modification. These findings highlight microalgal EPS as a bioresource for sustainable nanomaterial synthesis. Future research should refine biosynthesis conditions for industrial scalability and explore applications in antimicrobial coatings, drug delivery, and wastewater treatment, reinforcing microalgae-driven nanotechnology as a viable green solution.

## Data Availability

The original contributions presented in the study are included in the article/Supplementary material, further inquiries can be directed to the corresponding author.
