# Supplementary material for: Microalgae-mediated green synthesis of silver nanoparticles: a sustainable approach using extracellular polymeric substances from Graesiella emersonii KNUA204
Source: Front Microbiol. 2025 May 14;16:1589285. doi: 10.3389/fmicb.2025.1589285 (PMC12116571; doi:10.3389/fmicb.2025.1589285)
Supplement: Supplementary file 1 [file Data_Sheet_1.docx]

Supplementary Material


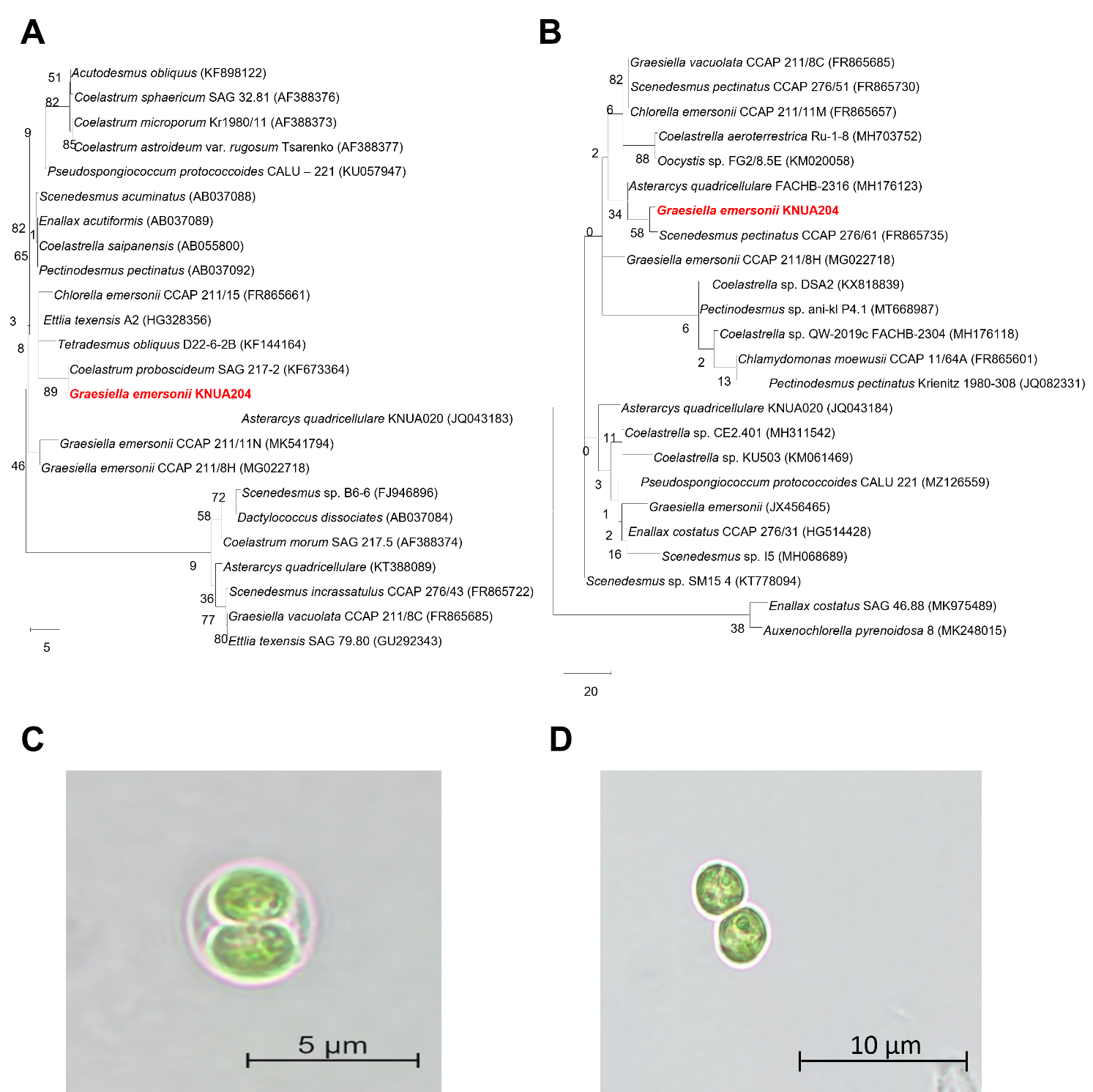


**Supplementary Figure 1.** Identification of *G. emersonii* collected from Ulleungdo Island. (A) Phylogenetic tree based on 18S rRNA sequences. (B) Phylogenetic tree based on ITS sequences. (C, D) Light microscopy images of *G. emersonii*.


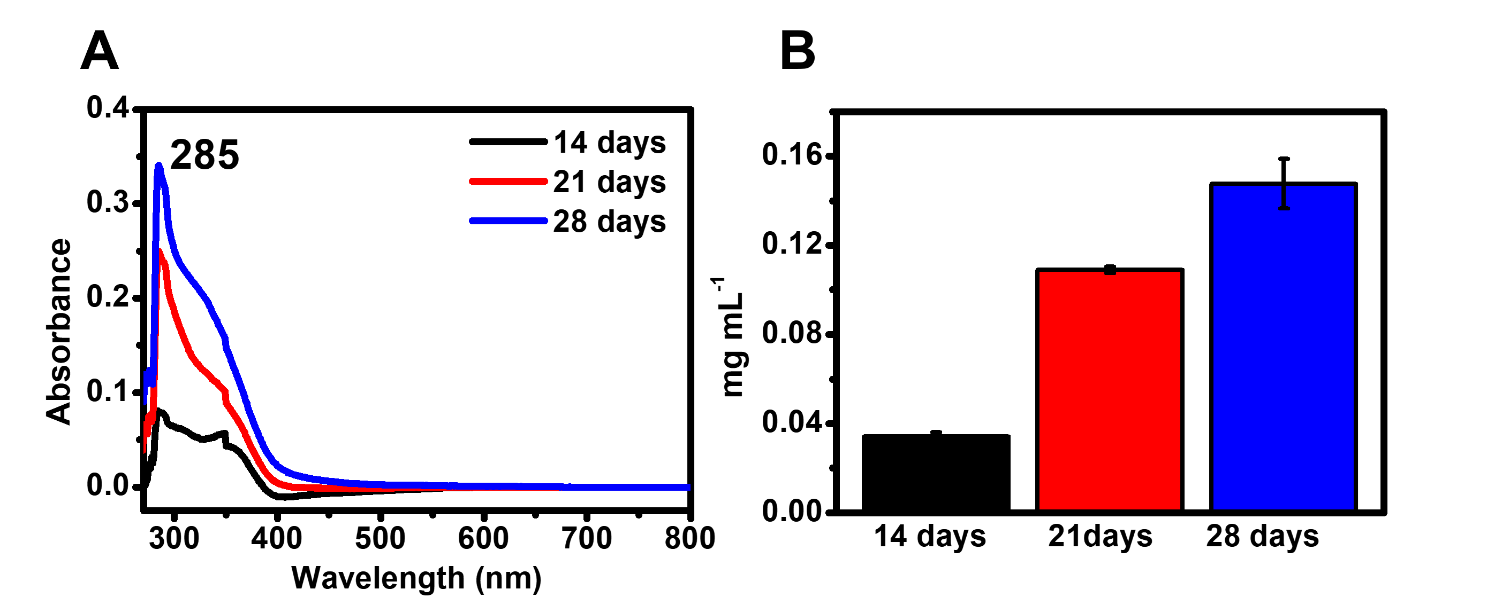


Supplementary Figure 2. Biosynthesis of AgNPs using supernatant of different pH.


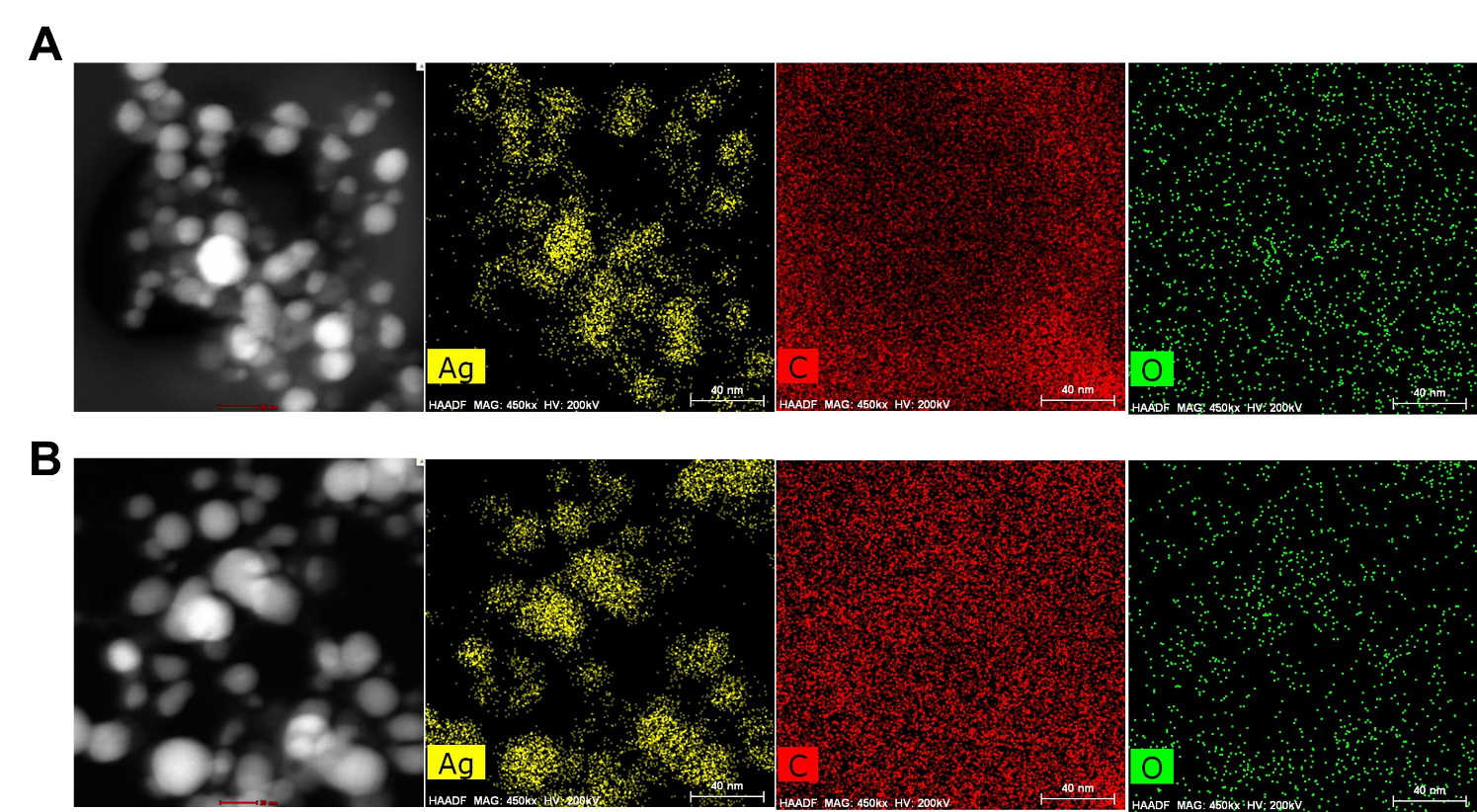


Supplementary Figure 3. Elemental mapping of (A) AgNPs and (B) Tetra-AgNPs.


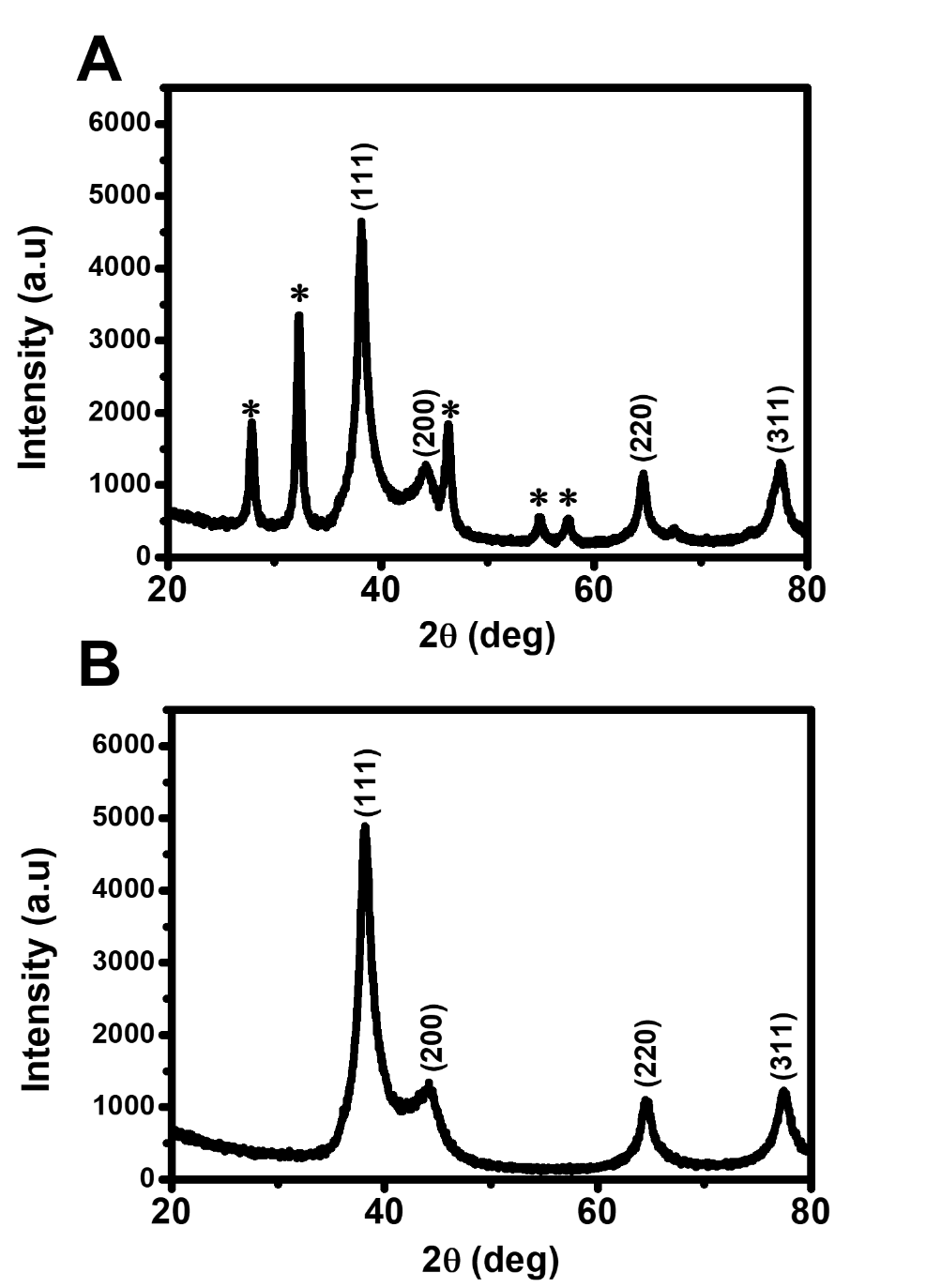


Supplementary Figure 4. XRD patterns for (A) AgNPs and (B)Tetra-AgNPs.


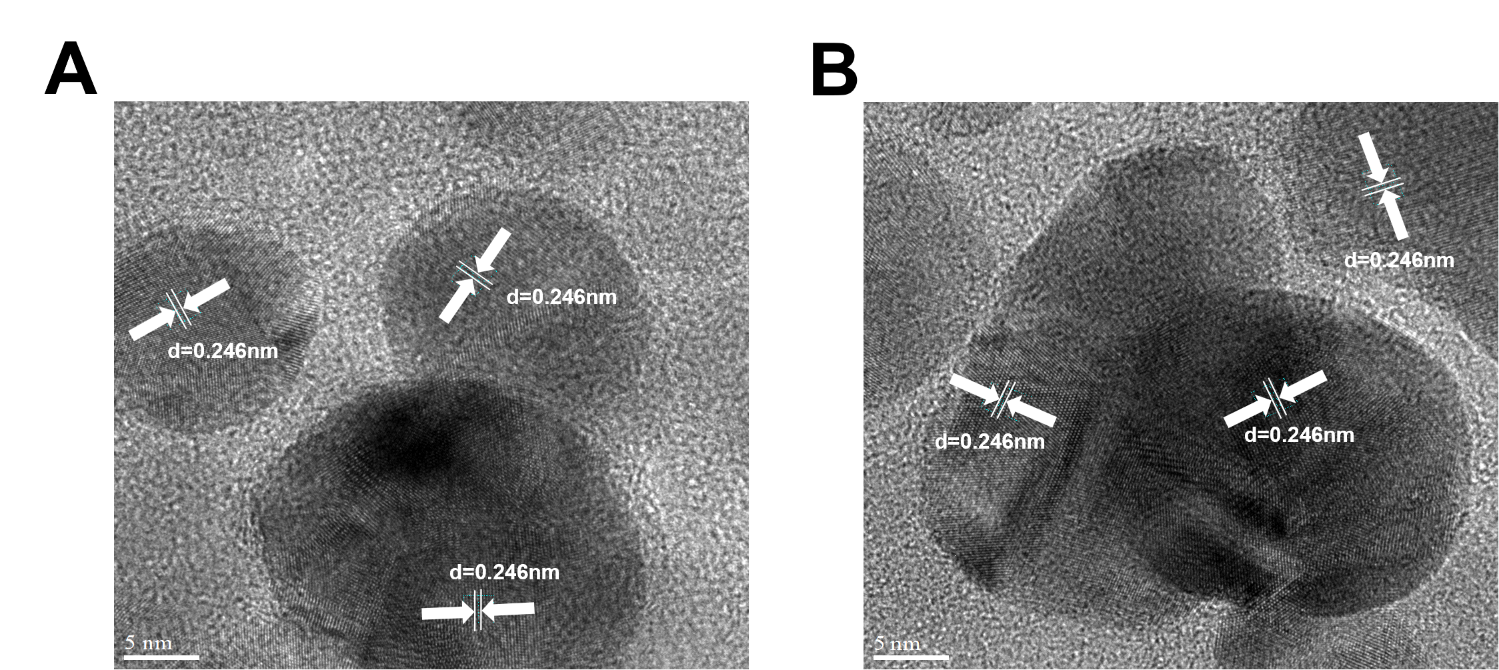


Supplementary Figure 5. TEM images of selected (A) AgNPs and (B) Tetra-AgNPs with d-spacing.


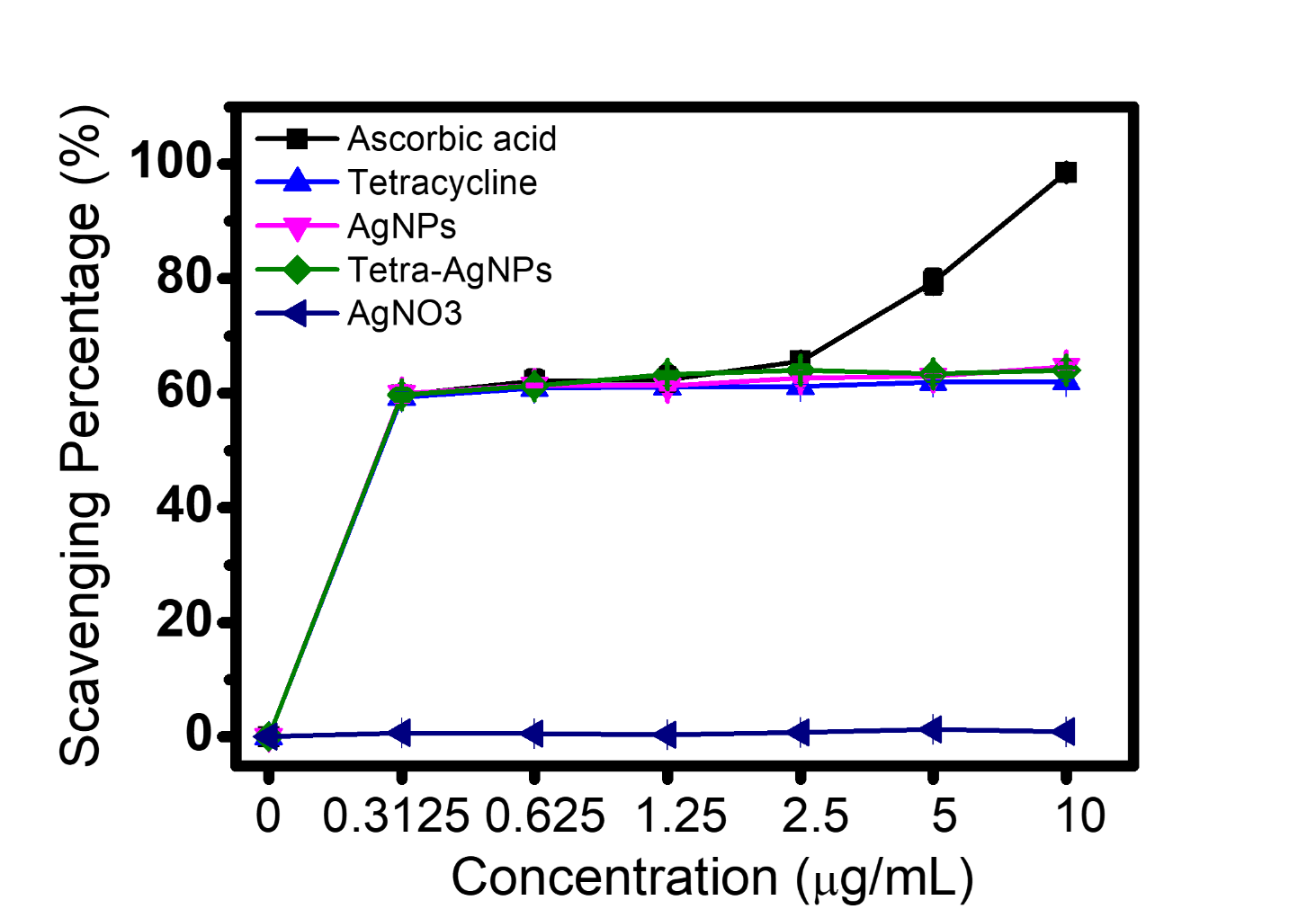


Supplementary Figure 6. Antioxidant activity of AgNPs and Tetra-AgNPs. Ascorbic acid was used as positive control for the DPPH assay.


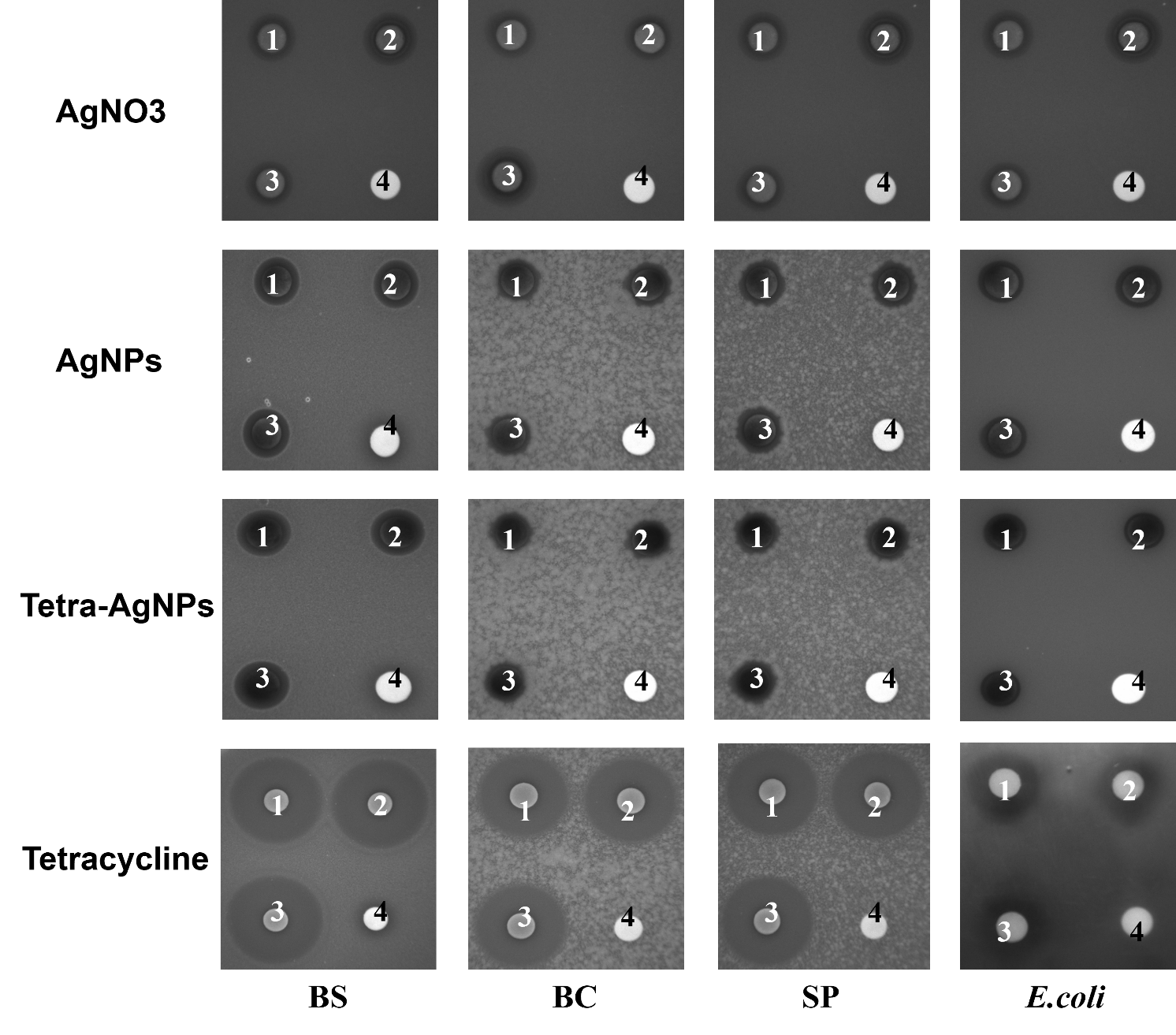


Supplementary Figure 7. Antibacterial activity of AgNPs and Tetra-AgNPs in solid media. Four bacterial stratins, *B. spizizenii* (BS), *B. cereus* (BC), *S. pasteuri* (SP), *E. coli* (E.coli) were used for the disc diffusion method. The number (1, 2, 3) indicated on the images represented triplicate experiments. The number 4 referred to as negative control (ultrapure water).


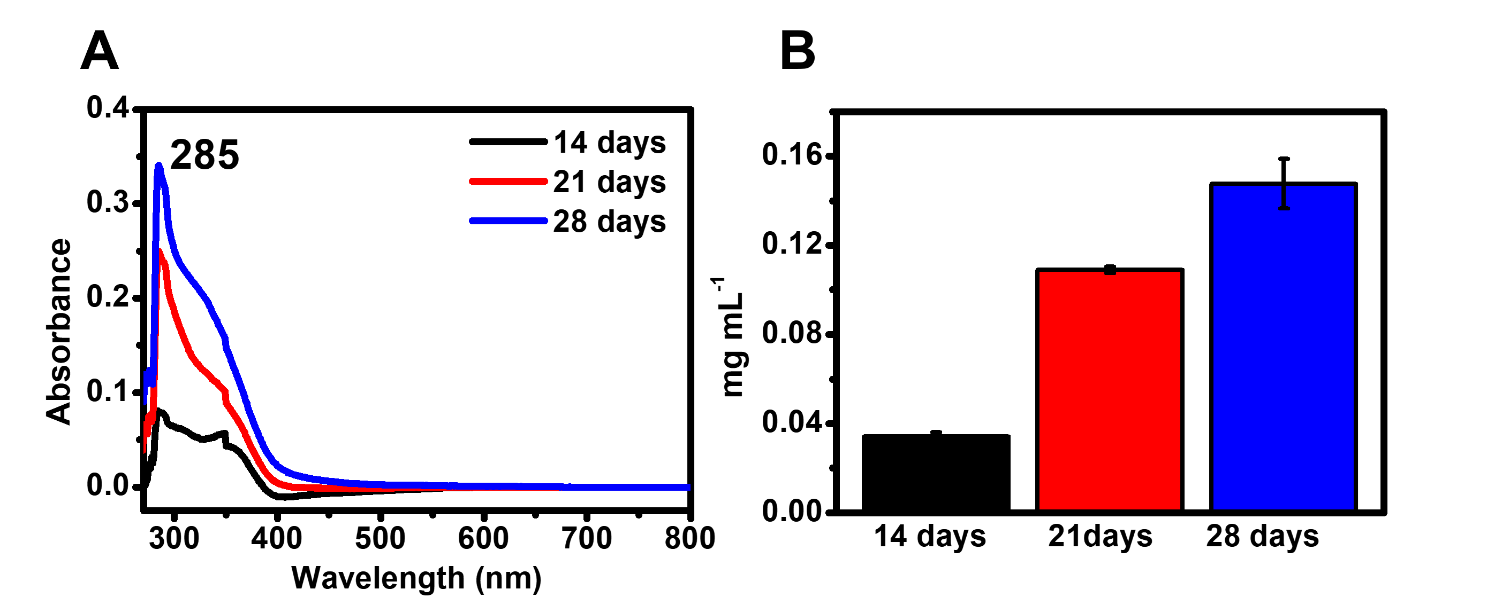


Supplementary Figure 8. (A) Absorbance spectrum of supernatant and (B) weight of soluble EPS of *G. emersonii*.

Supplementary Table 1. Comparative summary of silver nanoparticles (AgNPs) biosynthesized using different microalgal species. The table presents key characteristics including nanoparticle size, morphology, zeta potential (as a measure of colloidal stability), and antibacterial activity. This comparison highlights the performance and diversity of microalgae-mediated AgNP synthesis, providing context for the evaluation of *Graesiella emersonii* KNUA204 used in the present study.

| Microalgae Species | AgNP Size (nm) | Shape | Stability (Zeta Potential) | Antibacterial Activity | Reference |
| --- | --- | --- | --- | --- | --- |
| *Chlorella minutissima* | ~73 nm (average diameter) | Spherical | –21.2 mV (highly stable) | Broad-spectrum inhibition of Gram(+) and Gram(–) bacteria | Kumar *et al.*, 2024 |
| *Chlorella sorokiniana* | ~79 nm (optimized conditions) | Spherical | –23.7 mV | Effective against *E. coli* (zone ~20 mm) and *S. aureus* (~15 mm) | Kumar & Bharadvaja, 2022 |
| *Coelastrella aeroterrestrica* | ~14–15 nm (average diameter) | Hexagonal (dominant) with some quasi-spherical | –33 mV (highly stable) | Strong activity; e.g. *S. aureus* zone ~19 mm, *E. coli* ~15 mm | Hamida *et al.*, 2022 |
| *Arthrospira platensis* | 15–100 nm (range) | Spherical | ~–20 mV (stable dispersion) | Notable antibacterial efficacy; zones: *E. faecalis* ~16 mm, *S. aureus* ~14 mm (at 800 μg/mL) | Salman *et al.*, 2024​ |
| *Oscillatoria* sp. | ~10 nm (average size) | Spherical | N/A (thermostable per TGA) | Broad antibacterial effect (inhibition zones up to 21 mm) | Adebayo-Tayo *et al.*, 2019 |
| *Haematococcus pluvialis* | 30–50 nm (TEM size range) | Quasi-spherical | ~–30 to –40 mV (optimized: –40.4 mV) | Inhibits *E. coli* growth (significant zone observed) | Boboescu *et al.*, 2024​ |

Kumar, L., Mohan, L., Anand, R., & Bharadvaja, N. (2024). *Chlorella minutissima*-assisted silver nanoparticles synthesis and evaluation of its antibacterial activity. *Systems Microbiology and Biomanufacturing*, *4*(1), 230-239.

Kumar, L., & Bharadvaja, N. (2022). Biosynthesis, characterization, and evaluation of antibacterial and photocatalytic dye degradation activities of silver nanoparticles biosynthesized by *Chlorella sorokiniana*. *Biomass Conversion and Biorefinery*, 1-11.

Hamida, R. S., Ali, M. A., Almohawes, Z. N., Alahdal, H., Momenah, M. A., & Bin-Meferij, M. M. (2022). Green synthesis of hexagonal silver nanoparticles using a novel microalgae *Coelastrella aeroterrestrica* strain BA_Chlo4 and resulting anticancer, antibacterial, and antioxidant activities. *Pharmaceutics*, *14*(10), 2002.

Obaid, Z. H., Juda, S. A., Kaizal, A. F., & Salman, J. M. (2024). Biosynthesis of silver nano particles (AgNPs) from blue green algae (*Arthrospira platensis*) and their anti-pathogenic applications. *Journal of King Saud University-Science*, *36*(7), 103264.

Adebayo-Tayo, B., Salaam, A., & Ajibade, A. (2019). Green synthesis of silver nanoparticle using *Oscillatoria* sp. extract, its antibacterial, antibiofilm potential and cytotoxicity activity. *Heliyon*, *5*(10).

Savvidou, M. G., Kontari, E., Kalantzi, S., & Mamma, D. (2023). Green synthesis of silver nanoparticles using the cell-free supernatant of *Haematococcus pluvialis* culture. *Materials*, *17*(1), 187.
